# Lentiviral vector design using alternative RNA export elements

**DOI:** 10.1186/1742-4690-4-38

**Published:** 2007-06-05

**Authors:** Taekeun Oh, Ali Bajwa, Guangfu Jia, Frank Park

**Affiliations:** 1 Department of Medicine, Kidney Disease Center, Medical College of Wisconsin, 8701 Watertown Plank Road, Milwaukee, WI, USA; 2 Department of Physiology, Medical College of Wisconsin, 8701 Watertown Plank Road, Milwaukee, WI, USA; 3 Department of Internal Medicine, Chungbuk National University Hospital, Cheongju, South Korea; 4 Department of Medicine, Gene Therapy Program, Louisiana State University Health Sciences Center, 533 Bolivar St., New Orleans, LA, USA

## Abstract

**Background:**

Lentiviral vectors have been designed with complex RNA export sequences in both the integrating and packaging plasmids in order to co-ordinate efficient vector production. Recent studies have attempted to replace the existing complex rev/RRE system with a more simplistic RNA export system from simple retroviruses to make these vectors in a rev-independent manner.

**Results:**

Towards this end, lentiviral transfer plasmids were modified with various cis-acting DNA elements that co-ordinate RNA export during viral production to determine their ability to affect the efficiency of vector titer and transduction in different immortalized cell lines in vitro. It was found that multiple copies of the constitutive transport element (CTE) originating from different simian retroviruses, including simian retrovirus type 1 (SRV-1) and type-2 (SRV-2) and Mason-Pfizer (MPV) could be used to eliminate the requirement for the rev responsive element (RRE) in the transfer and packaging plasmids with titers >10^6 ^T.U./mL (n = 4–8 preparations). The addition of multiple copies of the murine intracisternal type A particle, the woodchuck post-regulatory element (WPRE), or single and dual copies of the simian CTE had minimal effect on viral titer. Immortalized cell lines from different species were found to be readily transduced by VSV-G pseudotyped lentiviral vectors containing the multiple copies of the CTE similar to the findings in HeLa cells, although the simian-derived CTE were found to have a lower infectivity into murine cell lines compared to the other species.

**Conclusion:**

These studies demonstrated that the rev-responsive element (RRE) could be replaced with other constitutive transport elements to produce equivalent titers using lentivectors containing the RRE sequence *in vitro*, but that concatemerization of the CTE or the close proximity of RNA export sequences was needed to enhance vector production.

## Background

Gene transfer applications have been widely developed over the past decade using various viral and non-viral vectors, including lentivectors based on the human immunodeficiency virus [1]. Significant modifications have been made in the HIV-based vectors since the initial findings by Naldini *et al*. [2] that have allowed the vector to perform effectively in the absence 5 out of the 9 wild-type HIV genes, specifically *vif*, *vpr*, *vpu*, *nef *and *tat *[3-7]. Continual efforts in the past few years have attempted to further delete wild-type HIV sequences in the lentiviral transfer (integrating) and packaging vector to enhance vector titer and transgene expression. One of the main regulatory genes, *rev*, has remained in many of the advanced lentivectors, and this protein is known to bind to its *cis*-acting DNA element, *rev*-responsive element (RRE). This rev/RRE system is important to the efficient transport of unspliced viral RNA genomes from the nucleus into the cytoplasm to properly assemble the lentivector particles [8]. There has been recent work to assess the use of alternative RNA export sequences from simple retroviruses to replace the more complex rev/RRE system, including individual copies of simian retrovirus [9,10] and Mazon-Pfizer [11,12] constitutive transport element (CTE). The results in terms of viral titer varied from extremely poor [11,12] to moderately high [9]. Moreover, the previous studies produced the vector using RRE-dependent packaging systems. Earlier work by Wodrich *et al*. [13] demonstrated that *gag *polyprotein (Pr55) expression can be significantly elevated if multiple copies of the Mason-Pfizer CTE were incorporated into the *gag-pol *expression plasmid compared to a single copy of the CTE, which may circumvent the requirement of the *rev*/RRE post-transcriptional control systems in vector production.

At present, there have been no attempts to produce lentiviral vectors with multiple copies of CTE in either the transfer (integrating) or packaging plasmids. For this reason, the goal of the present study was to develop alternative versions of lentivectors using concatemer RNA export sequences from simple retroviruses to determine the dispensability of the complex rev/RRE system to generate high-titer lentiviral vectors. In our study, we compared the role of the woodchuck post-regulatory element (WPRE) as well as the efficacy of various constitutive transport elements (CTE) from simian retrovirus type 1 (SRV-1) and type-2 (SRV-2), Mason-Pfizer retrovirus, and the murine intracisternal type A particle element (IAPE). In all, these basic vector issues should help to better understand the role of RNA export sequences in the effective production of lentiviral vectors and their functionality *in vitro*.

## Results

### Construction of advanced lentivector transfer plasmids using different *cis*-acting DNA elements

**Figure 1:**
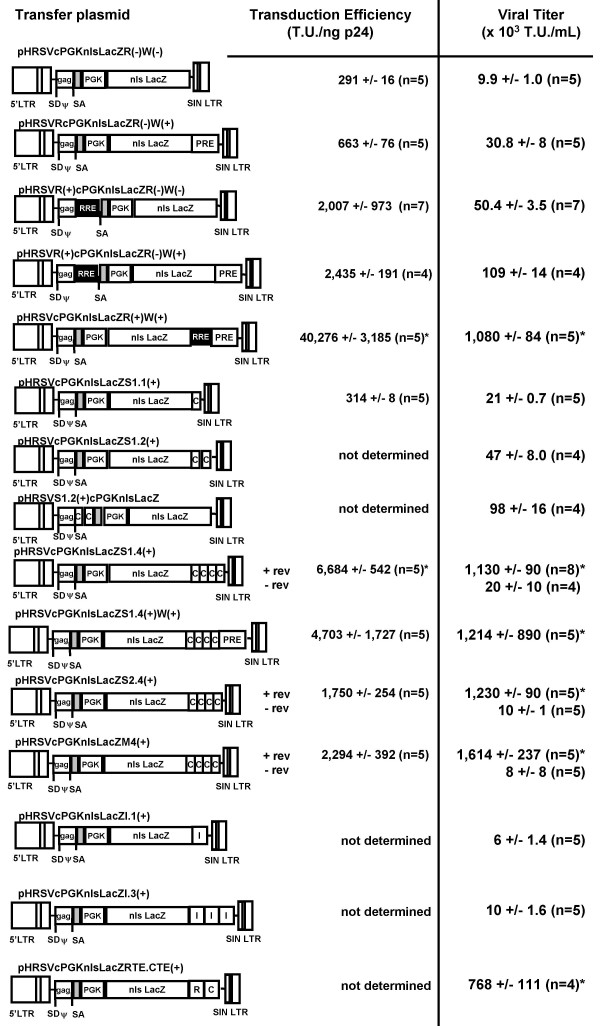
**Schematic of a panel of transfer plasmids containing different *cis*-acting DNA elements: Role on viral titer and transduction efficiency**. The basic lentiviral transfer plasmid used in this experiment were derivatives of the pHRSVcPGKnlsLacZR(-)W(-), which contains the murine PGK promoter driving the expression of the nuclear localized lacZ gene (nlsLacZ). The central polypurine tract sequence (cppt; GREY box) was cloned into all of the lentiviral transfer plasmids to maximize transduction efficiency. Splice donor (SD and acceptor (SA) sites were included in every transfer plasmid. Viral titer was determined by end-point dilution on HeLa cells, and p24 Gag protein levels were determined by ELISA. C = constitutive transport element; WPRE = woodchuck post-regulatory element; RRE = rev-responsive element; SIN LTR = 3' self-inactivating long-terminal repeat. n = 4–8 different lentiviral preparations/transfer plasmid. * p < 0.05 difference between the basic lentiviral vector (pHRSVcPGKnlsLacZR(-)W(-).

As shown in Figure 1, lentivector transfer plasmids were cloned with a variety of RNA export/transport elements to determine their ability to generate lentivectors titers equivalent to the existing rev/RRE system.

The basic lentivector transfer plasmid used in this study contained the phosphoglycerokinase (PGK) promoter driving the expression of nuclear localized bacterial lacZ gene. In addition, the cis-acting DNA element known as the central polypurine tract sequence (or cppt) from the *pol *gene was included 5' to the PGK promoter due to its well-known ability to enhance viral transduction efficiency [4-6]. Splice donor (SD) and acceptor (SA) sites were included in every transfer vector plasmid. The viral titer of this basic lentivector was 9.9 +/- 1.0 × 10^3 ^T.U./mL. The WPRE did not significantly enhance the viral titer (30.8 +/- 8 × 10^3 ^T.U./mL; n = 4), which was expected since its main functional role is to stabilize expressed transcripts [14]. The insertion of the RRE into the transfer plasmid 5' to the PGK promoter [pHRSVR(+)cPGKnlsLacZR(-)W(-)] increased the viral titer to 50.4 +/- 3.5 × 10^3 ^T.U./mL (n = 5), and there appeared to be a co-operative effect on viral titer when the RRE was included into the lentivector transfer plasmid 5' to the PGK promoter with the WPRE near the 3' self-inactivating long-terminal repeat (SIN LTR; 109 +/- 14 × 10^3 ^T.U./mL; n = 4). The incorporation of the RRE into our lentivector transfer plasmid was position-dependent, since we found that the placement of the RRE near the 3' end of the lentivector resulted in a significant enhancement of viral titer to 1,080 +/- 84 × 10^3 ^T.U./mL, which was ~20-fold higher than the RRE sequence in the 5' region of the vector.

To determine whether RNA export sequences from other simple retroviruses could replace the RRE to maximize lentivector titer, we cloned four different constitutive transport elements (CTEs) from simian retrovirus type 1 (SRV-1) and 2 (SRV-2), Mazon-Pfizer retrovirus (MPV), and murine intracisternal A-type particles (IAP). A single copy insertion of a minimal CTE from SRV-1 near the 3' SIN LTR of the lentivector transfer plasmid resulted in no detectable change in viral titer (11 +/- 3 × 10^3 ^T.U./mL) compared to the lentivector transfer plasmid, pHRSVcPGKnlsLacZR(-)W(-). Incorporation of a second CTE resulted in a slight increase in the lentivector titer to 47 +/- 8 × 10^3 ^T.U./mL (n = 3). A position-effect was observed if the dual CTE complex was cloned into the 5' end of the lentivector transfer plasmid adjacent to the *gag *gene fragment, since the titer increased by ~2-fold to 98 +/- 16 × 10^3 ^T.U./mL (n = 4).

As we increased the number of SRV-1 CTE to 4 head-to-tail copies, we calculated a significant increase (p < 0.05) in viral titer to 1,130 +/- 90 × 10^3 ^T.U./mL (n = 8). The increase in viral titer with the four copies of the SRV1 CTE was ~3-log orders higher than the vectors containing only one copy of the SRV-1 CTE. Comparatively, lentivector titers were observed to be in the 10^6 ^T.U. range even with the replacement of the SRV-1 CTEs with either SRV-2 or MPV CTE, i.e., 1,230 +/- 90 × 10^3 ^T.U./mL (n = 5) and 1,614 +/- 237 × 10^3 ^T.U./mL (n = 5), respectively.

**Figure 2:**
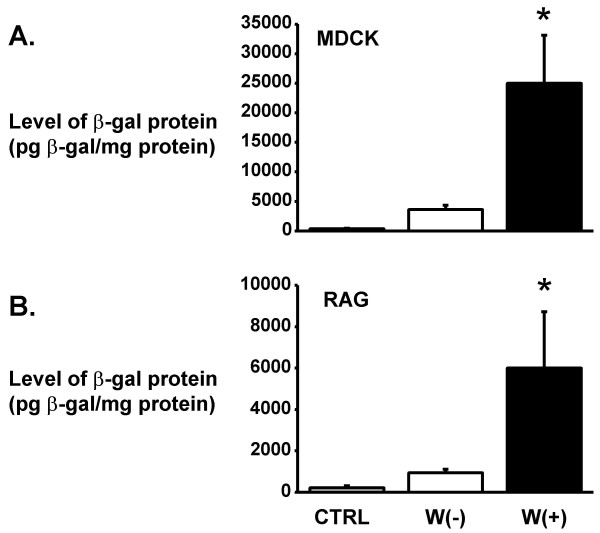
**Role of the WPRE on β-gal protein expression in cell lines following lentiviral vector transduction**. Canine (MDCK; Fig. 2A) and murine (RAG; Fig. 2B) immortalized cells were transduced with lentiviral vectors in the presence and absence of the WPRE in the transfer plasmid. The lentiviral transfer plasmid used in this experiment was the pHRSVcPGKnlsLacZS1.4(+) and pHRSVcPGKnlsLacZS1.4(+)W(+). The MDCK (MOI 3) and RAG (MOI 1) were transduced with the VSV-G pseudotyped lentiviral vectors and 48 hours later, the proteins were isolated and assayed for β-gal protein by ELISA. C = central polypurine tract sequence; PGK = murine phosphoglycerokinase promoter; nlsLacZ = nuclear localized lacZ gene; S1.4(+) = 4 copies of the SRV-1 CTE; W(-) = no WPRE; W(+) = WPRE. n = 3–5 different samples/lentiviral preparation. * p < 0.01 difference between the different groups.

Insertion of the WPRE 3' to the 4 multimeric copies of the SRV-1 did not result in a marked change in the viral titer. Since the WPRE is known to enhance the stability of mRNA transcripts [14], we examined whether the incorporation of the WPRE in combination with the multimeric CTE would affect the steady-state protein levels following transduction (Figure 2). Total β-gal protein levels following the transduction of MDCK cells (at a MOI 3) using the pHRSVcPGKnlsLacZS1.4(+)W(+) transfer vector resulted in an increase of 6.5-fold compared to the pHRSVcPGKnlsLacZS1.4(+) transfer vector (i.e., 24,990 +/- 8,134 versus 3,611 +/- 778 pg β-gal/mg protein). Similar increases in β-gal protein levels were found in RAG cells using a MOI 1 (6,002 +/- 2,721 versus 932 +/- 185 pg β-gal/mg protein; n = 5). These results demonstrated that the WPRE in the context of bacterial lacZ gene could enhance the level of transgene expression, which is similar to previous studies in our lab using second-generation lentivector systems [15].

### Role of the murine intracisternal type A element (IAPE) on viral titer

A CTE-related element known as a RNA transport element (RTE), which is located within an introns of the osteocalcin-related gene, and previously found to functionally replace the rev/RRE during HIV replication [16], was cloned in close proximity to the minimal SRV-1 CTE and found to significantly increase the viral vector titer to 768 +/- 111 × 10^3 ^T.U./mL compared to the SRV-1 CTE alone (11 +/- 3 × 10^3 ^T.U./mL).

Another murine IAP element (IAPE), which was located in the *pol *gene of MIA14 [17], was cloned into the lentivector transfer plasmid resulting in no significant change in functional titer (6.0 +/- 1.4 × 10^3 ^T.U./mL) compared to the RNA export minus lentivector system (9.9 +/- 1.0 × 10^3 ^T.U./mL). Inserting two additional copies of the murine IAPE only increased the viral titer to 1.0 +/- 1.63 × 10^4 ^T.U./mL, and this appeared to be orientation-dependent since the IAPE inserted into the opposite direction resulted in no detectable X-gal positive HeLa cells (data not shown). There would appear to be a variable effect by different IAPE on their functional ability to replace the rev/RRE system to generate lentiviral vector stocks.

### Lentivector transduction into immortalized kidney cell lines from different species

**Figure 3:**
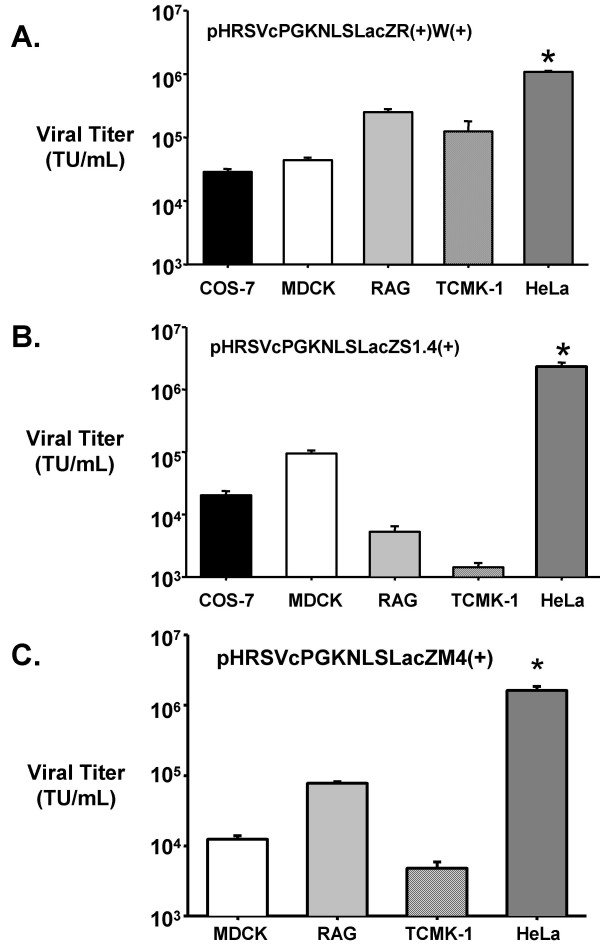
***In vitro *analysis of viral transduction into a panel of immortalized cell lines**. Advanced lentiviral vectors were used for transduction into various immortalized kidney-based cell lines. COS-7 (African green monkey), MDCK (dog), TCMK-1 and RAG (mouse) were transduced with VSV-G pseudotyped lentiviral vectors produced in 293T cells using three different transfer plasmids, pHRSVcPGKnlsLacZR(+)W(+) (Fig. 3A), pHRSVcPGKnlsLacZS1.4(+) (Fig. 3B) and pHRSVcPGKnlsLacZM4(+) (Fig. 3C). n = 5 different lentiviral preparations/cell line. * p < 0.05 difference between HeLa versus all of the other immortalized cell lines.

The optimal lentivectors as determined in HeLa cells were used to transduce a variety of kidney-derived cell lines *in vitro*. As shown in Figure 3, the lentivectors that contained the RRE in the 3' end were found to transduce 1.08 +/- 0.08 × 10^6 ^T.U./mL in HeLa cells. It was found that all of the immortalized cell lines from a number of different species were not as efficient on viral uptake, integration, and expression of the lacZ gene using the VSV-G pseudotyped advanced lentivectors. All of the cells lines derived from dogs (MDCK), monkeys (COS-7), and mice (TCMK-1 and RAG) had similar levels of transduction with the highest transduction found in the RAG cell line (2.52 +/- 0.28 × 10^5 ^T.U./mL).

On the other hand, lentivectors containing multiple copies of the CTE (see Figure 3B) in the transfer plasmid transduced COS-7 and MDCK at similar efficiencies as the lentivectors containing the RRE. However, there was a significantly lower lentivector titer in the murine cell lines (TCMK-1 and RAG). Similar results were obtained using lentivectors containing the multimer copies of the MPV CTE (Fig. 3C). One of the main reasons for the lower viral titer on the murine cell lines, particularly TCMK-1, may be due to the origin of the CTE, which is simian in nature, and so cell lines derived from large animal species may have cellular factor(s) that are not normally present in rodents.

### Construction of different lentivector packaging plasmids

**Figure 4:**
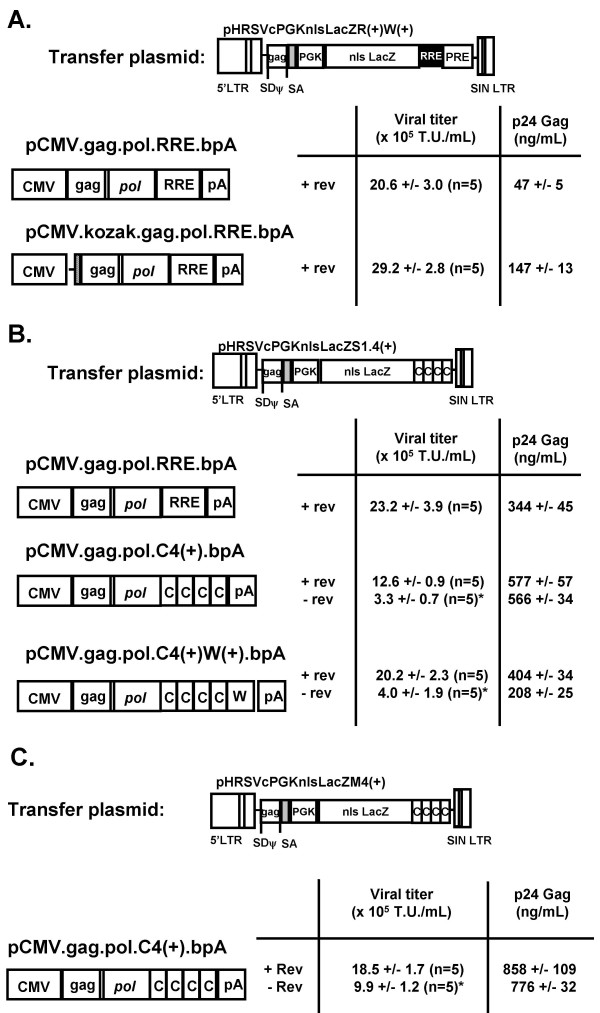
**Schematic and viral titer of different lentiviral vector packaging constructs**. Lentiviral vectors were produced using different transfer plasmids containing RRE (Fig. 4A) or multiple copies of SRV-1 (Fig. 4B) or MPV CTE (Fig. 4C). Viral titer was determined by end-point dilution using X-gal stained HeLa cells and p24 Gag protein ELISA. HATCHED bars = Kozak sequence (CCACCATGG); W = woodchuck post-regulatory element; C = constitutive transport element; pA = bovine growth hormone poly(A) signal; RRE = rev-responsive element; SD = splice donor; SA = splice acceptor. * p < 0.05 difference between lentiviral vectors produced with or without the *rev *protein.

Minimal packaging plasmids containing the *gag-pol *genes from HIV-1 were cloned as described in the Materials and Methods, in which the DNA sequences attributed to the expression of the viral accessory genes were deleted. As shown in Figure 4, there did not appear to be any significant difference in viral titer using different *cis*-acting DNA elements in the packaging constructs. Various transfer plasmids were examined to determine their compatibility with different packaging plasmid containing the RRE or multiple sequences of the SRV-1 CTE. Our study found that there was a marked difference in p24 Gag protein levels depending on the combination of transfer and packaging plasmid used for lentivector production as the levels ranged from 47 +/- 5 ng/mL (n = 5; pCMV.gag.pol.RRE.bpA) to 858 +/- 109 ng/mL [n = 5; pCMV.gag.pol.C4(+).bpA]. p24 Gag protein measurements by ELISA has been one common method to titer lentivector preparations, and generally, the functional viral titer of lentiviral vectors is dependent upon the p24 Gag protein levels. For this reason, we cloned the consensus Kozak sequence (CCACCATGG) in front of the Gag-pol genes in the packaging construct to enhance translation of p24 Gag protein production. As shown in Figure 4A, the viral titer in HeLa cells were similar (between 2–4 × 10^6 ^T.U./mL) following the production of a lentiviral vector containing the same transfer plasmid, but using two different *gag-pol *constructs to express the structural and packaging genes. The interesting finding was that inclusion of the Kozak sequence (pCMV.Kozak.gag.pol.RRE.bpA) resulted in an approximate 3-fold increase of p24 Gag protein to 147 +/- 12.5 ng/mL, but the functional viral titer as determined by end-point dilution did not markedly change compared to the packaging plasmid without the Kozak sequence. Interestingly, the p24 Gag levels were 2–3-fold higher using a transfer plasmid containing multiple CTE elements with either a RRE-containing packaging plasmid or a WPRE-containing packaging plasmid. The elevated p24 Gag protein levels did not appear to affect the viral titers in a positive way. These results demonstrate that the functional titer is not necessarily a function of the p24 Gag protein levels provided that a minimal threshold of gag protein is produced, and that several different factors, such as optimizing translation start site and other cis-acting DNA elements may play a role in producing high titer vector.

Another interesting aspect from our experiments was the RRE-dependent increase in vector titer even in the absence of the RRE in both the transfer and packaging plasmids. The co-transfection of the *rev*-expressing plasmid during vector production resulted in a consistent increase in viral titer by 2–5 fold depending on the transfer plasmid used in these studies (Figure 4B and 4C). There was definite *rev*-dependence in producing even moderate levels of functional lentiviral vector when the RRE was cloned within the packaging plasmid (Figure 1). There was no difference in the *rev*-dependence on increasing viral titer regardless of the source of the CTE (i.e., MPV or SRV-1), but the main issue is that vector titer using packaging plasmids with multiple copies of the CTE replacing the RRE could produce relatively high titer vector, but that the expression of *rev *would further increase the viral titer. The mechanism is not known, but it could be due to the significant secondary structure that could form using the multimeric CTE, which could simulate the secondary structure of the RRE, or that rev protein indiscriminately binds to secondary structures or DNA elements in the vector to allow for efficient export of the unspliced gene products and viral genomes into the cytoplasmid for assembly.

## Discussion

The present study demonstrated the importance of multiple RNA export sequences to replace the *rev*/RRE system in the production of replication-defective lentiviral vectors.

The incorporation of alternative RNA export elements into the packaging plasmid would potentially eliminate the need for the expression of the *rev *gene during vector production. RNA export sequences, such as constitutive transport elements (CTE), are known to increase the viability of the RNA genome of simple and complex retroviruses by transporting unspliced genomic RNA from the nucleus to the cytoplasm for packaging and assembly into viral particles. For this reason, the incorporation of alternative RNA export elements into the packaging plasmid would potentially eliminate the need for the expression of the *rev *gene during vector production.

In our present study, however, we demonstrated that minimal lentivectors could be designed in which the RRE was replaced in both the transfer or packaging plasmid through the incorporation of multiple copies of different RNA export sequences, specifically the CTE from SRV-1, SRV-2 or MPV. Each of the concatemeric CTE sequences in the context of the lentiviral transfer vector resulted in relatively high viral titers (>10^6 ^T.U./mL) compared to lentiviral vectors containing individual copies of the CTE. Other *cis*-acting elements, such as the woodchuck post-regulatory element (WPRE), did not play a significant role in enhancing viral titer, but may have been involved in transcript stability [14] and/or transgene expression [15,18].

Consistent with previous studies using simple [18] and complex retroviral vectors [11,12], an individual copy of the CTE was unable to replace the RRE in enhancing the production of functional vector particles. There have been previous reports documenting the detection of functional lentivector titers [9,19] and HIV-1 replication [10,17,20] using individual copies of SRV-1 [9,10] and murine IAP [17,20] using a *rev*-dependent system. In these studies, the lentivector titers [9,19] and HIV-1 replication [10,20] were ~20- and ~10-fold lower, respectively, using the CTE compared to the RRE.

Early efforts to develop a complete *rev*-independent HIV-1 based vector system was achieved by Kotsopoulou *et al*. [21] who codon-optimized the *gag-pol *genes to minimize the genetic overlap between the transfer and packaging plasmids during vector production. However, the codon-optimized lentivector appeared to be dependent on the presence of *rev*, since viral titers would drop 20-fold in the absence of the *rev *protein. Coincidentally, the lentivector titers in the absence of *rev *(3–4 × 10^5 ^T.U.mL) in the Kotsopoulou *et al*. [21] study were similar to the titers in our present study without *rev *(i.e., our newly developed three-plasmid system) using the multimeric SRV-1 CTE-containing transfer constructs. In our study, we were capable of generating lentiviral titers in excess of 10^6 ^T.U./mL in a *rev*-independent manner using multiple copies of the MPV CTE. Although the mechanism by which the multimeric copies of the CTE produced higher vector titers were not determined in our study, it is likely that there was more efficient export of the unspliced viral RNA genome from the nuclei to the cytoplasm allowing for more effective packaging during assembly into a functional vector, which would be consistent with a previous study by Wodrich *et al*. [13].

Other RNA export sequences from murine origin known as intracisternal A-type particles (IAP) were examined for their function on viral titer. In our hands, we found that the insertion of a single RNA export element from IAP (IAPE) did not affect the viral titer even if multiple copies were included into the transfer plasmid. This contrasts with the previous studies by Wodrich *et al*. [17], who showed that the IAPE could significantly enhance Gag protein expression in a *rev*-independent manner, but apparently in the context of generating functional lentivector particles, the IAPE is incapable of sufficiently replacing the *rev*/RRE nor the CTE systems. On the other hand, a distinct murine IAP known as the RTE cloned in close proximity of an individual SRV-1 CTE has been shown to replace the *rev*/RRE system during HIV replication [22,23]. Consistent with these previous findings, we found that the RTE-CTE complex increased the lentiviral vector titer by ~37-fold compared to the lentiviral vector with only a single copy of the SRV-1 CTE (Figure 1). Interestingly, the incorporation of the RTE 5' to 4 copies of the MPV CTE (data not shown) did not affect the viral titer, which may be due to the saturation of the RNA export system resulting in a maximal amount of unspliced RNA for viral packaging and protein translation.

One important caveat to the replacement of the regulated *rev*/RRE system with multiple copies of the RNA export elements from simple retroviruses is the potential for intermolecular recombination during vector production due to the extremely high nucleotide homology of these elements (88–92% identical). Therefore, the use of different CTEs may not make a significant difference in minimizing recombination. However, we have performed replication-competency assays to measure for p24 Gag antigen by ELISA and have not seen any difference in RCL formation regardless of the combination of CTE elements compared to the widely used RRE-containing vector systems (data not shown), but more sensitive PCR based assays may discover intermolecular recombination at the genetic level.

## Conclusion

Our present study demonstrated that advanced lentivectors can be effectively produced using alternative RNA export sequences from simple retroviruses at fairly high titers (>10^6 ^T.U./mL) provided that they are incorporated as a concatemer or in close proximity with one another. These experiments define the importance of certain *cis*-acting DNA elements in the context of lentiviral vector production.

## Methods

### Construction of the 5' long terminal repeat (LTR) fused with the Rous Sarcoma Virus U5 region

The RSV U5 region was amplified from pRSV.hAAT.bpA (Yant *et al*., 2000; generously given to me by Dr. Stephen. R. Yant, Stanford, CA) and ligated into the *Sma*I site of pBS.HIVLTR to make pRSV.HIVLTR. The SV40 poly(A) signal was amplified by PCR and cloned into the novel *Pme*I site 5' to the RSV U5 region in pRSV.HIVLTR to make pSV40pA.RSV.HIVLTR, which was subsequently cloned into a lentiviral vector transfer plasmid through multiple standard cloning steps.

### Construction of transfer plasmids containing different *cis*-acting DNA elements

The basic lentiviral transfer plasmid, pHRSVcPGKnlsLacZR(-)W(-) contained the murine phosphoglycerokinase (mPGK) promoter driving the expression of the nuclear localized lacZ gene. The central polypurine tract sequence (cppt; Zennou *et al*., 2000) was cloned 5' to the mPGK promoter into the *Xho*I site. pHRSVcPGKnlsLacZR(-)W(+) was cloned by replacing the *Nde*I/*Nhe*I site from pHR'CMVlacZW(+)SIN into the *Nde*I/*Nhe*I site of pHRSVcPGKnlsLacZR(-)W(-). The pHRSVR(+)cPGKnlsLacZR(-)W(-) and pHRSVR(+)cPGKnlsLacZR(-)W(+) plasmids were cloned by inserting the *Not*I/*Xho*I fragment from pHR(+)cPGKnlsLacZR(-)W(+) into the *Not*I/*Xho*I site of pHRSVcPGKnlsLacZR(-)W(-) and pHRSVR(+)cPGKnlsLacZR(-)W(+), respectively. The pHRSVcPGKnlsLacZR(+)W(+) plasmid was produced by inserting the *Nde*I/*Nhe*I fragment from pHR(-)cCMVsollacZR(+)W(+) into pHRSVcPGKnlsLacZR(-)W(-).

### Construction of SRV-1 CTE-containing shuttle plasmids

Minimal 173 bp SRV-1 CTE was PCR amplified from pNL43R(-)Rev(-).S (generous gift from Dr. Barbara K. Felber, NCI-FCRDC, Frederick, Md) and cloned into the *Bam*HI site of pBS.bpA, which contained the bovine growth hormone poly(A) signal, to make pBS.CTE.bpA. Additional copies of the CTE (*Bam*HI/*Bgl*II fragment) were cloned into the *Bam*HI site to make pBS.CTE4.bpA. The WPRE was amplified from pBS.SK(+).WPRE.B11 (generously given to us by Thomas J. Hope, Univ. of Illinois, Champaign, Il) and cloned into the pBS.CTE4.bpA plasmid to make pBS.C4(+)W(+).bpA.

### Construction of SRV-1 CTE-containing transfer plasmids

pHRSVcPGKnlsLacZR(-)W(-) was digested to insert the CTE from pBS.CTE in order to make pHRSVcPGKnlsLacZS1.1(+). To make pHRSVcPGKnlsLacZS1.4(+), the pBS.C4(+) plasmid was digested with *Bam*HI and *Kpn*I and inserted into pHRSVcPGKnlsLacZR(-)W(-). The C4(+)W(+) fragment was cloned into the pHRSVcPGKnlsLacZR(-)W(-) to make pHRSVcPGKnlsLacZS1.4(+)W(+).

### Construction of SRV-2 CTE-containing transfer plasmids

Minimal 173 bp SRV-2 CTE was PCR amplified (generous gift from Dr. Barbara K. Felber, NCI-FCRDC, Frederick, Md) and cloned into the *Bam*HI site of pBS.bpA, which contained the bovine growth hormone poly(A) signal, to make pBS.S2.1(+).bpA. Additional copies of the SRV-2 CTE (*Bam*HI/*Bgl*II fragment) were cloned into the *Bam*HI site to make pBS.S2.4(+).bpA. To make pHRSVcPGKnlsLacZS2.4(+), the pBS.S2.4(+) plasmid was blunt digested, gel purified, and cloned into the *Pme*I site of pHRSVcPGKnlsLacZPmeI.

### Construction of MPV CTE-containing transfer plasmids

Minimal 174 bp MPV CTE was PCR amplified (generous gift from Dr. Barbara K. Felber, NCI-FCRDC, Frederick, Md) and cloned into the *Bam*HI site of pBS.bpA, which contained the bovine growth hormone poly(A) signal, to make pBS.M1(+).bpA. Additional copies of the MPV CTE (*Bam*HI/*Bgl*II fragment) were cloned into the *Bam*HI site to make pBS.M4(+).bpA. To make pHRSVcPGKnlsLacZM4(+), the pBS.M4(+).bpA plasmid was digested with *Bam*HI and *Kpn*I and inserted into pHRSVcPGKnlsLacZR(-)W(-).

### Construction of IAPE-containing transfer plasmids

pHR'CMVlacZSIN-18F was digested with *Kpn*I, blunted with Klenow fragment, and the minimal IAPE (isolated from 3-IAPE plasmid by PCR amplification; generous gift from Dr. H. Wodrich, Salk Institute, La Jolla, CA) was inserted to make pHR'CMVlacZI(+). This plasmid was subsequently digested with *Nde*I/*Nhe*I and the fragment containing the IAPE and 3' SIN LTR was cloned into pHRSVcPGKnlsLacZR(-)W(+) to make pHRSVcPGKnlsLacZI(+). Three copies of the IAPE were cloned into pBS-SK(-).IAPE3, and then digested with *Pme*I/*Sma*I for insertion into the novel *Pme*I site in pHR'CMVlacZ *Pme*ISIN to make pHR'CMVlacZI.3(+). Note that the IAPE3 was cloned in both the forward and reverse directions. This was subsequently cloned into pHRSVcPGKnlsLacZR(-)W(+) and the R(+)W(+) segment was replaced to make pHRSVcPGKnlsLacZI.3(+) in the sense and antisense directions.

### Construction of RTEm26-CTE-containing transfer plasmids

pHRSVcPGKnlsLacZPmeISIN was digested with *Pme*I, which was a novel site near the 3' SIN LTR. The pNLgagM26CTE (generous gift from Dr. B. K. Felber) was double digested with *Kpn*I/*Sal*I and blunted with T4 DNA polymerase for ligation into the *Pme*I site. The final construct was pHRSVcPGKnlsLacZRTEm26CTE(+). Another construct was cloned to replace the single copy of the SRV-1 CTE with a multimer of CTE (4 copies) from the MMPV CTE to make the final construct of pHRSVcPGKnlsLacZRTEM4(+).

### Construction of the packaging plasmid

pCMVΔR8.74 [3] was digested with *Xba*I and *Eco*RI, and an oligo linker containing novel *Bam*HI and *Pme*I sites was inserted into the packaging plasmid backbone. The *Bam*HI/*Pme*I fragment from pBS.RRE.bpA was inserted into pCMV.gag.pol.oligo to make pCMV.gag.pol.RRE.bpA. The pBS.C4(+).bpA plasmid was digested with *Bam*HI and *Pme*I, and this fragment was inserted into pCMV.gag.pol.oligo to make pCMV.gag.pol.C4(+).bpA. The packaging plasmid, pCMV.gag.pol.C4(+)W(+).bpA was made by inserting the C4(+)W(+)bpA fragment into the *Bam*HI/*Pme*I sites.

### PCR assay

Oligonucleotides were purchased from Integrated DNA Technologies (Coralville, IA). All PCR conditions were similar and used the following conditions: 3' at 94°C (initial melt) followed by 35 cycles of 94°C for 30 sec, 55°C for 30 sec, and 72°C for 10 sec. Final extension was performed at 72°C for 7 minutes. All sequences that were amplified and cloned into the vectors were sequenced.

### Cell lines

Kidney-derived cells lines from different species were obtained from ATCC, specifically LLC-PK_1 _(porcine), MDCK (canine), TCMK-1 (mouse), RAG (mouse) and 293T cells (human). COS-7 cells (African green monkey) were obtained from Dr. S. M. Lanier (Medical University of South Carolina, Charleston, SC).

### Envelope pseudotype plasmids

pMD.G is the envelope plasmid and encodes the vesicular stomatitis virus G protein as previously described [3].

### Lentiviral vector production

Modified second-generation lentiviral vectors were produced by transient triple-plasmid transfection of 293T cells as previously described [5,24,25]. The advanced third-generation lentiviral vectors were produced by a similar manner as the three-plasmid system, but the following amounts of plasmid DNA were used: 10 μg transfer plasmid, 6.5 μg packaging plasmid, 3.5 μg envelope plasmid and 5 μg rev-expressing plasmid (pRSV121; generous gift from Dr. T. J. Hope, University of Illinois). Conditioned media were collected at 48 hrs, filtered and frozen at -80°C. End-point dilution by X-gal staining was performed on HeLa cells when titering lacZ-expressing lentiviral vectors. p24 Gag protein ELISA was performed on all of the lentiviral vector preps using a commercially available kit (NEN). All lentiviral vectors were produced and titered at the same time to minimize variability from batch-to-batch preparations and titering conditions.

### β-gal ELISA

MDCK and RAG cells were plated at a density of 5 × 10^5 ^cells in 6-well dishes and infected with lentiviral vectors with [pHRSVcPGKnlsLacZS1.4(+)W(+)] or without the WPRE [pHRSVcPGKnlsLacZS1.4(+)]. RAG and MDCK cells were transduced at MOIs of 1 and 3, respectively, using the titered values from HeLa cells. The media was replaced 24 hours following the initial infection, and then the cells were collected 24 hours later. The cells were lysed using the reagents in the β-gal ELISA kit from Roche (Mannheim, Germany). The ELISA was performed as per the manufacturer's protocol and the plate was scanned using a Bio-Rad plate reader 680 (Hercules, CA) at 490 nm.

### Statistical analysis

The significance of differences between groups at the same dose of lentivirus was tested by a one-way ANOVA with the use of StatView 5.0 software (SAS Institute Inc., Cary, NC). If a probability value of P < 0.05 was obtained, the Tukey test was used for comparison of each individual group with the appropriate control groups.

## Competing interests

The author(s) declare that they have no competing interests.

## Authors' contributions

TO and AB were co-authors responsible for the cell culture production and viral titer analysis in Figures 1, 3 and 4.

GJ was responsible for the cell culture experiments to complete the data for Figure 2.

FP was responsible for the design of the experiment, cloning of all of the vectors, and writing of the manuscript.
